# Effect of Baduanjin exercise on cervical spondylosis: A protocol for the systematic review of randomized controlled trials

**DOI:** 10.1097/MD.0000000000024813

**Published:** 2021-03-26

**Authors:** Liang Zou, Chongfu Zhong, Xiaohui Xu, Fanjie Liu, Congan Wang, Bin Shi

**Affiliations:** a School of Acupuncture-Tuina, Shandong University of Traditional Chinese Medicine; b Affiliated Hospital of Back and Neck Pain, Shandong First Medical University; c The First Affiliated Hospital of Shandong University of Chinese Medicine, Jinan, Shandong, China.

**Keywords:** Baduanjin, cervical spondylosis, protocol, systematic review

## Abstract

**Background:**

The study aims to evaluate the effectiveness and safety of Baduanjin exercise for patients with cervical spondylosis (CS).

**Methods:**

We will retrieve a randomized controlled trial of Baduanjin for CS from the following electronic databases establishment to May 2021: The Cochrane Library, MEDLINE, EMBASE, Web of Science, Springer, World Health Organization International Clinical Trials Registry Platform (ICTRP), China National Knowledge Infrastructure (CNKI), Wan-fang database, Chinese Scientific Journal Database (VIP), Chinese Biomedical Literature Databases (CBM), and other databases. Two independent researchers will operate article retrieval, duplication removing, screening, quality evaluation, and data analyses by Review Manager (V.5.3.5). Meta-analyses, subgroup analysis, and/or descriptive analysis will be performed based on the included data conditions.

**Results:**

The results of this study will provide researchers in the field of CS with a current synthesis of high-quality evidence.

**Conclusion:**

This conclusion of this study will provide the evidence of whether Baduanjin is an effective and safe intervention for patients with CS.

**PROSPERO registration number:**

CRD42020211019.

## 1 Introduction

### 1.1 Description of the condition

Cervical spondylosis (CS) is a cervical chronic spine disc degenerarive pathology, which influence the vertebral bodies and cervical intervertebral discs. It may develop into bone spur production, protrusion of intervertebral disc, and spinal cord compression.^[1]^ Patients with degenerative cervical spondylosis may present with headaches, neck stiffness, numbness, neck and/or arm pain, neck and/or arm tingling, or a combination of these symptoms, which seriously affects people's quality of life.^[2]^ The etiological factors include age, sex, occupation, education levels, area, behavior, heredity, and sports-related activities.^[3]^ The Study found this disease was most common between the ages of 40 and 60,^[4,5]^ but the Epidemiological investigation shows that the incidence of CS is increasing and getting younger year by year.^[6]^ Treatment for CS includes conservative and surgical therapy. Conservative therapy consists of rest, immobilization of the cervical spine, drug therapy, and physical therapy.^[7]^ Surgery is expensive and not appropriate for every patient, and there are varying degrees of sides affects.^[8]^

### 1.2 Description and function of intervention

Baduanjin, one of the most common forms of Qigong exercise, dates back to the Chinese Song Dynasty (10th–13th century A.D). It is composed of 8 simple independent movements and characterized by slow movements, mental concentration and meditation, regulated breathing to achieve a harmonious flow of Qi in the body.^[9]^ In recent years, due to its effectiveness for keeping fit, ease in learning, and economy of exercising time, Baduanjin has become popular worldwide as a promising low-intensity, physical and mental exercise.^[10]^ Studies have shown that through Baduanjin exercise, can enhance the strength of neck and back muscles, enhance the flexibility and stability of neck and shoulder, and then maintain the stability of the cervical spine. Also can alleviate muscle spasm, improve bone structure, reduce pain, prevent muscle atrophy, restore and improve cervical motor function, prevent cervical joint stiffness, improve cervical blood circulation, promote inflammation of the anti-inflammatory.^[11–16]^

### 1.3 Why the review is important

Cervical spondylosis is very common disease that affects a large proportion of the population.^[17]^ Cervical spondylosis can cause a range of symptoms, such as dizziness, headaches, neck stiffness, numbness, shoulder pain, and even paralysis.^[18]^ Recent studies suggest that cervical is a risk factor for localized spinal cord lesions in multiple sclerosis.^[19]^ This disease can significantly reduce the quality of people's life, and patients with a heavy financial burden.^[5]^ In Chinese clinical trials, many treatments for patients with CS have drawn the method of Baduanjin treatment.^[20–22]^ However, the evidence was still limited based on nonstandard measurement, nonuniformed outcomes, subjectivity judgment, and other factors. Furthermore, no relevant review has been published, so it is necessary to conduct evidence-based review to evaluate the efficacy and safety of Baduanjin for CS in people, It is urgently needed to accomplish this review.

## 2 Methods

The systematic review protocol has been registered in the PROSPERO. The registration number: CRD42020211019. All steps of this systematic review will be performed according to the Cochrane Handbook (5.2.0).

### 2.1 Inclusion criteria for selection criteria

#### 2.1.1 Type of study

Randomized controlled trials (RCTs) and blinded research will be included. Published clinical trials that reported the efficacy and safety on Baduanjin for patients with CS will be included. RCTs that involve at least 1 Baduanjin related treatment to CS, and 1control treatment (or blank treatment) will be included. As there is a risk of interference with the outcome, nonrandomized controlled trials will be excluded. Observational, cohort, case-control, case series, qualitative and laboratory studies, and uncontrolled trials will be excluded.

#### 2.1.2 Types of patients

Patients who were diagnosed as CS, aged 18 to 55 years, will be included in the study, without limits on gender, weight, education, race, nationality and medical units.

#### 2.1.3 Types of interventions and comparisons

Interventions can be any type of Baduanjin. Multiple control interventions will be included: no treatment, placebo and other interventions (e.g., acupuncture, moxibustion, massage, cupping therapy, drugs and physical interventions). If its interventions and comparisons both contain Baduanjin, the study will be excluded. Interventions of Baduanjin combined with other therapies will be included, only if these combinations are compared to the other therapies semplice.

#### 2.1.4 Types of outcomes

Primary outcomes will include the neck disability index (NDI), neck pain questionnaire questionnaires. NDI is widely used to assess the functional status and treatment effect of patients with various types of CS, with high reliability and validity. The scale investigated the general conditions of patients and described their own pain. Secondary outcomes will include Patient Satisfaction Scale and, and side effects of Baduanjin.

### 2.2 Search methods for identification of studies

#### 2.2.1 Electronic searches

We will retrieve a randomized controlled trial of Baduanjin for CS from the following electronic databases establishment to May 2021; Cochrane Library, MEDLINE, EMBASE, Web of Science, Springer, World Health Organization International Clinical Trials Registry Platform (ICTRP), China National Knowledge Infrastructure (CNKI), Wan-fang database, Chinese Scientific Journal Database (VIP), Chinese Biomedical Literature Databases (CBM), and other databases. All published RCTs about this topic will be included. Exemplary search strategy of MEDLINE is listed in Table 1, terms are conform to medical subject heading (MeSH). According to the difference of databases, keywords may combined with free words and comprehensive search will be performed.

**Table 1:** MEDLINE search strategy.

#1 MeSH Major Topic: cervical spondylosis
#2 MeSH Major Topic: cervical spondylopathy
#3 MeSH Major Topic: cervical osteoarthtitis
#4 MeSH Major: Baduanjin exercise
#5 MeSH Major: Baduanjin
#6MeSH Major: BDJ
#7MeSH Major: BDJE
#8MeSH Major: Qigong
#9 MeSH Major: eight section brocades
#11 #1 or #2 or #3
#12 #4 or #5 or #6 #7 or #8 or #9
#13 #11 and #12

### 2.3 Data collection and analysis

#### 2.3.1 Selection of studies

**Figure 1:**
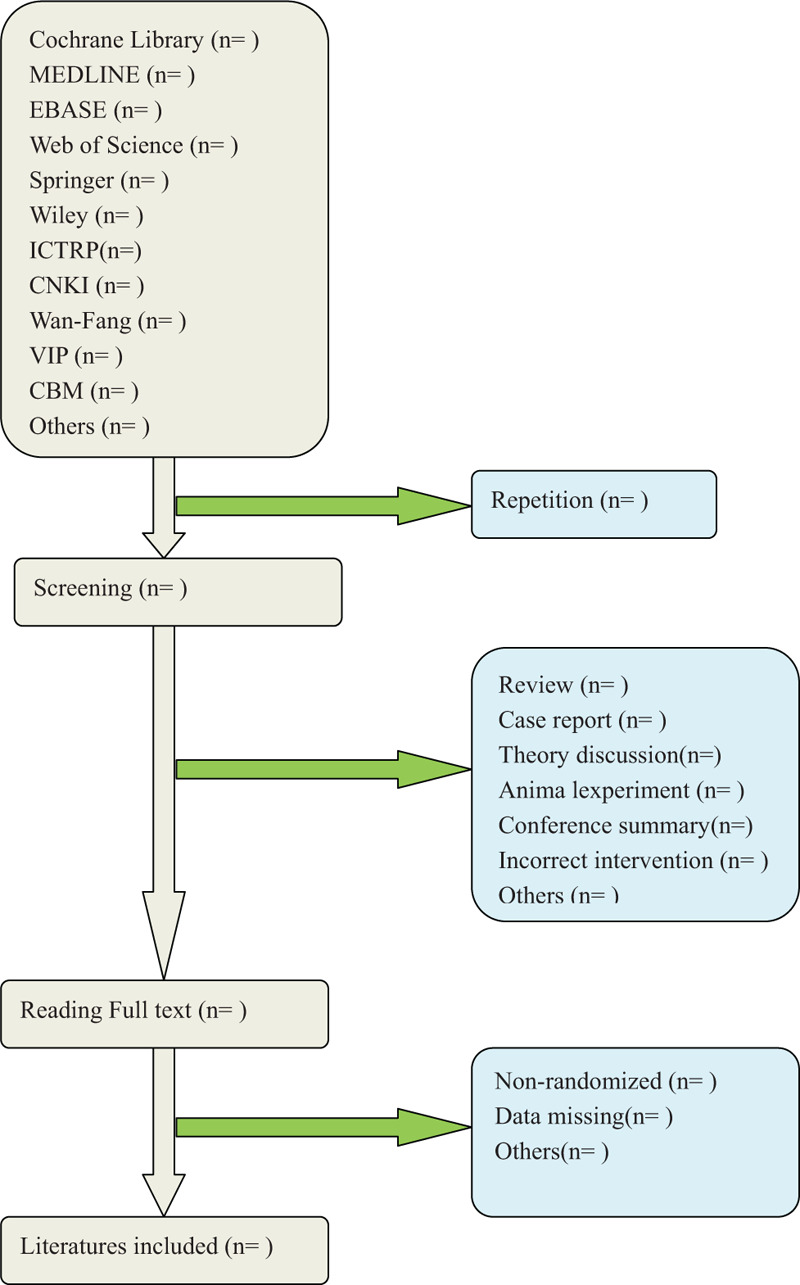
Flow diagram of studies identified.

Two authors (LZ and XHX) will select clinical trials depending on inclusion criteria. After the title and abstract are screened, literatures that are not related and do not meet the criteria will be excluded. Screening operation will flow the diagram of Figure 1. If the full literatures are unable to obtained or related data is incomplete, we will contact the corresponding author. Third-party experts will be consulted to determine the selection divergence.

#### 2.3.2 Assessment and quality of included studies

Two authors (CFZ and FJL) will evaluate quality of included articles and assess the risk of bias based on Cochrane Handbook 5.2.0. Quality assessment of included studies contains randomized method, allocation concealment, blinding of participants and personnel, blinding of outcome assessment, completeness of outcome data, and selective reporting. Divergence of evaluation will also consult third-party experts.

#### 2.3.3 Data extraction

The authors (LZ and CFZ) plan to extract the data from the articles selected for inclusion, and to resolve differences in opinion through discussion with experts. Data will be recorded onto an electronic form, including categories for basic information about the studies (numbing, the first author last name and the year the study was published, and the contact information for the corresponding author), the sample sizes and grouping methods used, participant characteristics including age and gender, expressed as mean additions and subtractions above and below standard deviation and the percentages, and details of the intervention methods involved, including treatment time, the selection of acupoints, treatment efficacy, treatment cycles, side effects, and follow-up.

#### 2.3.4 Measures of treatment effect

Two authors (LZ and XHX) will perform analysis independently and then cross-check treatment effect with Review Manager 5.3.5. Dichotomous data will be presented by risk ratio (RR) with 95% confidence intervals (CIs). Continuous data will be presented by mean difference (MD) or standard mean difference (SMD) with 95% CI. Other binary data will be changed into the RR form for analysis.

#### 2.3.5 Dealing with missing data

As there is possibility of missing data in literatures, we will contact the corresponding authors by email or other contacts. If the missing data are unavailable, we will analysis the existing data that is supposed as random missing.

#### 2.3.6 Assessment of heterogeneity

The heterogeneity of studies will be evaluated by Q-test and *I*^2^ statistic with RevMan5.3.5. The following criteria will be used: *I*^*2*^ < 50% will be deemed as low heterogeneity; *I*^*2*^ between 50% and 75% will be considered as moderate heterogeneity; *I*^*2*^ > 75% will be considered as high heterogeneity.

#### 2.3.7 Assessment of reporting bias

Publication bias and other reporting biases will be assessed by creating funnel plots. Symmetric funnel plots indicate low risk of bias, while dissymmetry ones may indicate high risk.

#### 2.3.8 Data synthesis

A meta-analysis or descriptive analysis will be performed, based on the intervention methods, the measurement methods, and heterogeneity levels, etc. If clinical and methodological heterogeneity are low, the fixed-effect model will be applied by merger analysis; the random-effects model will be applied by merger analysis when heterogeneity indicates a moderate level. If, however, a significant level of heterogeneity is found, a descriptive analysis will be performed instead.

#### 2.3.9 Subgroup analysis

Subgroup analysis will be performed based on the findings from the data synthesis, and if the heterogeneity is found to have been caused by particular features of the included studies (e.g., the intervention methods [type, time, and cycle] and the measurement methods used in the clinical trials), subgroup analysis will be conducted relevant to these categories.

## 3 Discussion

The incidence of CS is very high in the present society. In many cases, surgical or medical treatment is not necessary. As a non-invasive external therapy, Baduanjin is widely used in CS in China due to its simplicity, convenience and low cost. In recent years, there have been more and more clinical reports on the treatment of CS, but high quality trail is still insufficient. This review will begin when necessary trails are meeting. In order to give compelling evidence and better guide in clinic practice, all actions of this review will be performed according to Cochrane Handbook 5.2.0.

## Author contributions

**Investigation:** Xiaohui Xu.

**Methodology:** Liang Zou.

**Project administration:** Chongfu Zhong.

**Resources:** Chongfu Zhong, Xiaohui Xu.

**Software:** Bin Shi, Congan Wang.

**Validation:** Fanjie Liu.

**Visualization:** Fanjie Liu.

**Writing – original draft:** Liang Zou.

**Writing – review & editing:** Bin Shi, Fanjie Liu.
